# Correction: Genetic variation affects morphological retinal phenotypes extracted from UK Biobank optical coherence tomography images

**DOI:** 10.1371/journal.pgen.1009858

**Published:** 2021-10-18

**Authors:** Hannah Currant, Pirro Hysi, Tomas W. Fitzgerald, Puya Gharahkhani, Pieter W. M. Bonnemaijer, Anne Senabouth, Alex W. Hewitt, UK Biobank Eye and Vision Consortium, International Glaucoma Genetics Consortium, Denize Atan, Tin Aung, Jason Charng, Hélène Choquet, Jamie Craig, Peng T. Khaw, Caroline C. W. Klaver, Michiaki Kubo, Jue-Sheng Ong, Louis R. Pasquale, Charles A. Reisman, Maciej Daniszewski, Joseph E. Powell, Alice Pébay, Mark J. Simcoe, Alberta A. H. J. Thiadens, Cornelia M. van Duijn, Seyhan Yazar, Eric Jorgenson, Stuart MacGregor, Chris J. Hammond, David A. Mackey, Janey L. Wiggs, Paul J. Foster, Praveen J. Patel, Ewan Birney, Anthony P. Khawaja

The following information is missing from the Acknowledgments:

The International Glaucoma Genetics Consortium members are: Tin Aung, Kathryn P. Burdon, Ching-Yu Cheng, Jamie E. Craig, Angela J. Cree, Puya Gharahkhani, Christopher J. Hammond, Alex W. Hewitt, René Höhn, Pirro Hysi, Adriana I. Iglesias Gonzalez, Jost Jonas, Anthony Khawaja, Chiea-Cheun Khor, Caroline C.W. Klaver, Francesca Pasutto, Stuart MacGregor, David Mackey, Paul Mitchell, Aniket Mishra, Calvin Pang, Louis R. Pasquale, Francesca Pasutto, Henriette Springelkamp, Gudmar Thorleifsson, Unnur Thorsteinsdottir, Cornelia M. van Duijn, Ananth Viswanathan, Veronique Vitart, Janey L. Wiggs, Robert Wojciechowski, Tien Wong, Terri L. Young, Tanja Zeller.

The UK Biobank Eye and Vision Consortium members are: Naomi Allen, Tariq Aslam, Denize Atan, Sarah Barman, Jenny Barrett, Paul Bishop, Graeme Black, Catey Bunce, Roxana Carare, Usha Chakravarthy, Michelle Chan, Sharon Chua, Valentina Cipriani, Alexander Day, Parul Desai, Bal Dhillon, Andrew Dick, Alexander Doney, Cathy Egan, Sarah Ennis, Paul Foster, Marcus Fruttiger, John Gallacher, David Garway-Heath, Jane Gibson, Dan Gore, Jeremy Guggenheim, Chris Hammond, Alison Hardcastle, Simon Harding, Ruth Hogg, Pirro Hysi, Pearse A Keane, Peng Tee Khaw, Anthony Khawaja, Gerassimos Lascaratos, Thomas Littlejohns, Andrew Lotery, Phil Luthert, Tom MacGillivray, Sarah Mackie, Bernadette McGuinness, Gareth McKay, Martin McKibbin, Danny Mitry, Tony Moore, James Morgan, Zaynah Muthy, Eoin O’Sullivan, Chris Owen, Praveen Patel, Euan Paterson, Tunde Peto, Axel Petzold, Nikolas Pontikos, Jugnoo Rahi, Alicja Rudnicka, Jay Self, Panagiotis Sergouniotis, Sobha Sivaprasad, David Steel, Irene Stratton, Nicholas Strouthidis, Cathie Sudlow, Robyn Tapp, Caroline Thaung, Dhanes Thomas, Emanuele Trucco, Adnan Tufail, Veronique Vitart, Stephen Vernon, Ananth Viswanathan, Cathy Williams, Katie Williams, Jayne Woodside, Max Yates, Jennifer Yip, Yalin Zheng.
