# Supplementary material for: Genetic variation affects morphological retinal phenotypes extracted from UK Biobank optical coherence tomography images
Source: PLoS Genet. 2021 May 12;17(5):e1009497. doi: 10.1371/journal.pgen.1009497 (PMC8143408; doi:10.1371/journal.pgen.1009497)
Supplement: S1 Table — Results are presented as mean ± standard deviation. (PDF) [file pgen.1009497.s001.pdf]

	Total Topcon Population	Pass Filter	Fail Filter
Height (cm)	168.68 ±9.25	169.43 ±9.15	168.03 ±9.29
Age (years)	57 ±8	57 ±8	57 ±8
Weight (Kg)	78.12 ±16.02	78.48 ±15.83	77.81 ±16.18
Sex (m/f)	29713/34929	14837/16597	14876/18332
Refractive Error Left (Dioptres)	-0.32 ±2.73	0.23 ±1.49	-0.81 ±3.42
Refractive Error Right (Dioptres)	-0.38 ±2.73	0.19 ±1.48	-0.88 ±3.41
Glaucoma (present/absent)	6388/60210 (10.61%)	2751/28314 (9.72%)	3637/31896 (11.40%)
