# Supplementary material for: Genetic variation affects morphological retinal phenotypes extracted from UK Biobank optical coherence tomography images
Source: PLoS Genet. 2021 May 12;17(5):e1009497. doi: 10.1371/journal.pgen.1009497 (PMC8143408; doi:10.1371/journal.pgen.1009497)
Supplement: S2 Table — A1 is the effect allele. (PDF) [file pgen.1009497.s002.pdf]

SNP	Chr	BP	A1	A2	AF	MTAG effect size	RNFL effect size	GCIPL effect size	MTAG p-value	RNFL p-value	GCIPL p-value	MTAG SE	RNFL SE	GCIPL SE
rs72739513	1	203080149	A	G	0.04	0.72	0.37	0.72	8.88E-09	1.05E-04	8.88E-09	0.13	0.10	0.13
rs12998032	2	159095496	C	T	0.44	0.30	0.08	0.30	6.97E-10	2.12E-02	6.97E-10	0.05	0.04	0.05
rs13010692	2	48800667	C	T	0.32	0.22	0.22	0.09	6.72E-09	6.72E-09	0.09	0.04	0.04	0.05
rs2271758	2	172701157	G	T	0.59	-0.22	-0.22	-0.20	1.34E-09	1.34E-09	3.63E-05	0.04	0.04	0.05
rs79833181	2	15666802	C	T	0.02	0.86	0.86	0.57	1.55E-09	1.55E-09	2.43E-03	0.14	0.14	0.19
rs980772	2	145442190	T	G	0.67	-0.21	-0.21	0.03	4.62E-08	4.62E-08	0.60	0.04	0.04	0.05
rs13083522	3	3270368	G	A	0.78	0.31	0.08	0.31	4.51E-08	0.08	4.51E-08	0.06	0.04	0.06
rs149831820	3	77192591	C	T	0.06	-0.42	-0.42	-0.31	2.53E-08	2.53E-08	1.48E-03	0.07	0.07	0.10
rs17279437	3	45814094	A	G	0.11	-0.77	-0.31	-0.77	7.81E-24	1.23E-07	7.81E-24	0.08	0.06	0.08
rs62252355	3	69572006	C	T	0.20	-0.37	-0.37	-0.15	2.17E-16	2.17E-16	1.22E-02	0.05	0.05	0.06
rs66511946	4	184932935	G	A	0.40	-0.36	-0.11	-0.36	2.15E-13	4.36E-03	2.15E-13	0.05	0.04	0.05
rs17421627	5	87847586	G	T	0.07	0.97	-0.14	0.97	8.09E-27	4.87E-02	8.09E-27	0.09	0.07	0.09
rs2004187	5	2612747	C	A	0.60	0.25	0.25	0.29	1.43E-11	1.43E-11	2.77E-09	0.04	0.04	0.05
rs13215351	6	84313801	T	A	0.25	-0.33	-0.06	-0.33	1.36E-09	0.12	1.36E-09	0.05	0.04	0.05
rs527871768	6	39419992	A	G	0.01	1.16	1.16	0.71	3.37E-10	3.37E-10	3.43E-03	0.19	0.19	0.24
rs9398171	6	108983527	T	C	0.71	0.50	0.23	0.50	7.51E-22	1.45E-08	7.51E-22	0.05	0.04	0.05
rs11762530	7	46630602	C	G	0.59	0.53	0.07	0.53	3.45E-28	4.77E-02	3.45E-28	0.05	0.04	0.05
rs12719025	7	51100190	G	A	0.46	0.30	0.04	0.30	3.09E-10	0.29	3.09E-10	0.05	0.04	0.05
rs35001871	7	50975439	C	G	0.29	0.32	0.19	0.32	1.17E-09	2.98E-06	1.17E-09	0.05	0.04	0.05
rs73348111	7	50364291	C	T	0.01	1.12	1.12	0.66	7.15E-10	7.15E-10	5.70E-03	0.18	0.18	0.24
rs115520750	8	108739734	T	G	0.01	1.12	1.12	0.82	2.54E-10	2.54E-10	4.51E-04	0.18	0.18	0.23
rs13271359	8	109114426	T	C	0.26	-0.44	-0.44	-0.02	5.89E-26	5.89E-26	0.75	0.04	0.04	0.05
rs376067714	8	109141863	G	A	0.18	-0.48	-0.48	-0.11	1.28E-18	1.28E-18	0.13	0.05	0.05	0.07
rs4871827	8	121061879	A	G	0.33	-0.29	-0.12	-0.29	7.41E-09	2.00E-03	7.41E-09	0.05	0.04	0.05
rs6989495	8	74230223	T	G	0.33	0.23	0.23	0.14	1.27E-09	1.27E-09	4.33E-03	0.04	0.04	0.05
rs118031671	9	10521068	G	T	0.01	0.93	0.93	0.76	2.53E-09	2.53E-09	2.21E-04	0.16	0.16	0.21
rs2787394	9	103007414	T	C	0.41	-0.28	-0.06	-0.28	8.64E-09	0.09	8.64E-09	0.05	0.04	0.05
rs10762201	10	70040111	G	A	0.76	0.46	0.46	0.15	1.05E-26	1.05E-26	6.72E-03	0.04	0.04	0.06
rs181211282	10	102746829	A	G	0.03	0.67	0.67	0.24	1.12E-08	1.12E-08	0.11	0.12	0.12	0.15
rs1947075	10	49741135	T	C	0.64	-0.21	-0.21	-0.19	2.60E-08	2.60E-08	1.14E-04	0.04	0.04	0.05
rs1042602	11	88911696	A	C	0.37	-0.36	-0.36	-0.33	3.96E-22	3.96E-22	2.10E-11	0.04	0.04	0.05
rs12574166	11	69291285	T	C	0.15	0.28	0.28	0.24	2.82E-08	2.82E-08	3.92E-04	0.05	0.05	0.07
rs2008905	11	17184623	T	C	0.42	-0.36	-0.16	-0.36	6.81E-14	1.92E-05	6.81E-14	0.05	0.04	0.05
rs5442	12	6954864	A	G	0.07	-0.69	-0.08	-0.69	2.36E-13	0.27	2.36E-13	0.09	0.07	0.09
rs10140252	14	74528023	T	G	0.16	0.53	0.53	0.49	1.05E-25	1.05E-25	1.39E-13	0.05	0.05	0.07
rs1254276	14	60847001	T	C	0.39	-0.28	-0.28	-0.10	7.52E-14	7.52E-14	3.24E-02	0.04	0.04	0.05
rs146652416	14	29907103	G	A	0.03	0.62	0.62	0.45	4.46E-08	4.46E-08	2.44E-03	0.11	0.11	0.15
rs17095953	14	59719393	A	G	0.24	-0.28	-0.28	-0.08	1.76E-10	1.76E-10	0.14	0.04	0.04	0.06
rs35337422	14	104407243	C	A	0.15	0.37	-0.20	0.37	3.50E-08	1.28E-04	3.50E-08	0.07	0.05	0.07
rs1470108	15	89153744	A	C	0.34	0.24	0.24	0.21	7.09E-10	7.09E-10	3.77E-05	0.04	0.04	0.05
rs1800407	15	28230318	T	C	0.08	-0.60	-0.36	-0.60	3.19E-12	4.08E-08	3.19E-12	0.09	0.07	0.09
rs117304899	16	15055042	G	C	0.02	0.92	0.92	0.75	1.74E-09	1.74E-09	1.82E-04	0.15	0.15	0.20
rs117300236	17	44753350	G	A	0.72	-0.25	-0.25	-0.30	5.57E-09	5.57E-09	1.24E-07	0.04	0.04	0.06
rs7503894	17	79583473	C	T	0.65	0.56	0.37	0.56	2.49E-29	3.58E-22	2.49E-29	0.05	0.04	0.05
rs143330165	20	7154672	T	C	0.01	1.04	1.04	0.41	1.97E-08	1.97E-08	0.09	0.19	0.19	0.24
rs7277632	21	47327542	G	A	0.72	0.34	0.09	0.34	1.20E-10	3.56E-02	1.20E-10	0.05	0.04	0.05
