# Supplementary material for: Genetic variation affects morphological retinal phenotypes extracted from UK Biobank optical coherence tomography images
Source: PLoS Genet. 2021 May 12;17(5):e1009497. doi: 10.1371/journal.pgen.1009497 (PMC8143408; doi:10.1371/journal.pgen.1009497)
Supplement: S3 Table — References [1] Gharahkhani, P. et al. Genome-wide meta-analysis identifies 127 open-angle glaucoma loci with consistent effect across ancestries. Nature Communications 12 (2021). URL https://pubmed.ncbi.nlm.nih.gov/33627673/. (PDF) [file pgen.1009497.s003.pdf]

SNP	Risk allele	Other allele	Chr	Position	Nearest gene	OR for POAG	P-value
rs1254276	T	C	14	60847001	SIX6	1.18	2.03E-37
rs117300236	G	A	17	44753350	NSF	0.93	1.82E-05
rs5442	A	G	12	6954864	GNB3	1.11	9.97E-05
rs11762530	C	G	7	46630602	IGFBP3	1.04	1.52E-03
rs2787394	T	C	9	103007414	INVS	0.97	8.44E-03
rs1042602	A	C	11	88911696	TYR	1.03	1.47E-02
rs2008905	T	C	11	17184623	PIK3C2A	0.97	2.35E-02
rs7503894	C	T	17	79583473	NPLOC4	0.97	2.56E-02
rs17279437	A	G	3	45814094	SLC6A20	0.95	2.87E-02
rs10762201	G	A	10	70040111	ATOH7	0.97	2.95E-02
rs9398171	T	C	6	108983527	FOXO3	1.03	3.79E-02
rs17421627	G	T	5	87847586	LINC00461	1.05	0.07
rs2004187	C	A	5	2612747	IRX2	1.02	0.14
rs13271359	T	C	8	109114426	RSPO2	1.03	0.18
rs73348111	C	T	7	50364291	IKZF1	1.08	0.19
rs17095953	A	G	14	59719393	DAAM1	1.03	0.24
rs35337422	C	A	14	104407243	TDRD9	1.02	0.25
rs1470108	A	C	15	89153744	AEN	1.02	0.27
rs115520750	T	G	8	108739734	ANGPT1	1.07	0.37
rs79833181	C	T	2	15666802	NBAS	1.05	0.37
rs62252355	C	T	3	69572006	FRMD4B	1.01	0.39
rs12574166	T	C	11	69291285	LINC02747	0.99	0.49
rs13010692	C	T	2	48800667	STON1-GTF2A1L	1.01	0.55
rs143330165	T	C	20	7154672	LINC01428	1.07	0.57
rs72739513	A	G	1	203080149	ADORA1	0.98	0.58
rs117304899	G	C	16	15055042	NA	1.05	0.62
rs12719025	G	A	7	51100190	COBL	1.01	0.63
rs2271758	G	T	2	172701157	SLC25A12	0.99	0.63
rs980772	T	G	2	145442190	TEX41	0.99	0.64
rs181211282	A	G	10	102746829	MRPL43	0.98	0.67
rs4871827	A	G	8	121061879	DEPTOR	1.01	0.69
rs13083522	G	A	3	3270368	CRBN	0.99	0.69
rs66511946	G	A	4	184932935	STOX2	1.00	0.74
rs118031671	G	T	9	10521068	PTPRD	1.01	0.77
rs10140252	T	G	14	74528023	BBOF1	1.01	0.78
rs12998032	C	T	2	159095496	CCDC148	1.00	0.80
rs1800407	T	C	15	28230318	OCA2	0.99	0.81
rs13215351	T	A	6	84313801	SNAP91	1.00	0.86
rs146652416	G	A	14	29907103	FOXG1	0.99	0.86
rs6989495	T	G	8	74230223	RDH10	1.00	0.87
rs7277632	G	A	21	47327542	PCBP3	1.00	0.92
rs1947075	T	C	10	49741135	ARHGAP22	1.00	0.99
rs149831820	C	T	3	77192591	ROBO2	1.00	0.99
