# Supplementary material for: Genetic variation affects morphological retinal phenotypes extracted from UK Biobank optical coherence tomography images
Source: PLoS Genet. 2021 May 12;17(5):e1009497. doi: 10.1371/journal.pgen.1009497 (PMC8143408; doi:10.1371/journal.pgen.1009497)
Supplement: S4 Table — Comparison between effect size and p-values from meta-analysed inner retinal GWAS (labelled “MTAG”), and GWAS of the GCC thickness in the Rotterdam study (labelled “Rotterdam”). (PDF) [file pgen.1009497.s004.pdf]

SNP	MTAG effect size	MTAG p-value	Rotterdam effect size	Rotterdam p-value
rs1042602	-0.36	3.96E-22	-0.77	6.71E-03
rs10762201	0.46	1.05E-26	0.10	0.78
rs117300236	-0.25	5.57E-09	-0.03	0.94
rs11762530	0.53	3.45E-28	0.25	0.39
rs1254276	-0.28	7.52E-14	-0.20	0.48
rs12574166	0.28	2.82E-08	0.21	0.59
rs12719025	0.30	3.09E-10	0.99	5.89E-04
rs12998032	0.30	6.97E-10	0.22	0.44
rs13010692	0.22	6.72E-09	0.54	0.08
rs13083522	0.31	4.51E-08	0.03	0.93
rs13215351	-0.33	1.36E-09	-0.04	0.91
rs1470108	0.24	7.09E-10	0.34	0.27
rs149831820	-0.42	2.53E-08	0.27	0.69
rs17279437	-0.77	7.81E-24	-0.79	0.12
rs17421627	0.97	8.09E-27	1.62	2.21E-03
rs1800407	-0.60	3.19E-12	-1.12	0.16
rs1947075	-0.21	2.60E-08	-0.07	0.80
rs2004187	0.25	1.43E-11	0.31	0.29
rs2008905	-0.36	6.81E-14	0.07	0.81
rs2271758	-0.22	1.34E-09	-0.51	0.07
rs2787394	-0.28	8.64E-09	-0.36	0.21
rs35001871	0.32	1.17E-09	0.36	0.25
rs35337422	0.37	3.50E-08	0.24	0.55
rs4871827	-0.29	7.41E-09	-0.87	4.49E-03
rs5442	-0.69	2.36E-13	0.38	0.46
rs62252355	-0.37	2.17E-16	-0.27	0.43
rs66511946	-0.36	2.15E-13	-0.51	0.11
rs6989495	0.23	1.27E-09	1.00	9.50E-04
rs7277632	0.34	1.20E-10	1.35	2.25E-05
rs7503894	0.56	2.49E-29	0.57	0.06
rs9398171	0.50	7.51E-22	0.69	2.55E-02
rs980772	-0.21	4.62E-08	-0.23	0.43
