# Supplementary material for: Genetic variation affects morphological retinal phenotypes extracted from UK Biobank optical coherence tomography images
Source: PLoS Genet. 2021 May 12;17(5):e1009497. doi: 10.1371/journal.pgen.1009497 (PMC8143408; doi:10.1371/journal.pgen.1009497)
Supplement: S5 Table — Comparison between effect size and p-values from meta-analysed inner retinal GWAS (labelled “MTAG”), and GWAS of the RNFL and GCIPL in the Raine Study (labelled “Raine RNFL” and “Raine GCIPL”). (PDF) [file pgen.1009497.s005.pdf]

SNP	MTAG effect size	MTAG p-value	Raine RNFL effect size	Raine RNFL p-value	Raine GCIPL effect size	Raine GCIPL p-value
rs1042602	-0.36	3.96E-22	-0.20	0.11	-	-
rs10762201	0.46	1.05E-26	0.42	2.95E-03	-	-
rs115520750	1.12	2.54E-10	-0.66	0.39	-	-
rs117300236	-0.25	5.57E-09	-0.15	0.28	-	-
rs11762530	0.53	3.45E-28	-	-	0.78	8.00E-04
rs118031671	0.93	2.53E-09	-0.26	0.66	-	-
rs1254276	-0.28	7.52E-14	-0.24	0.05	-	-
rs12574166	0.28	2.82E-08	0.35	3.32E-02	-	-
rs12719025	0.30	3.09E-10	-	-	0.57	1.66E-02
rs12998032	0.30	6.97E-10	-	-	0.50	2.64E-02
rs13010692	0.22	6.72E-09	0.09	0.49	-	-
rs13083522	0.31	4.51E-08	-	-	-0.28	0.32
rs13215351	-0.33	1.36E-09	-	-	-0.27	0.30
rs143330165	1.04	1.97E-08	-1.81	0.21	-	-
rs146652416	0.62	4.46E-08	0.37	0.38	-	-
rs1470108	0.24	7.09E-10	0.10	0.43	-	-
rs149831820	-0.42	2.53E-08	-0.37	0.13	-	-
rs17279437	-0.77	7.81E-24	-	-	-1.76	1.53E-06
rs17421627	0.97	8.09E-27	-	-	0.58	0.20
rs1800407	-0.60	3.19E-12	-0.28	0.18	-0.49	0.23
rs181211282	0.67	1.12E-08	-0.19	0.65	-	-
rs1947075	-0.21	2.60E-08	0.02	0.86	-	-
rs2004187	0.25	1.43E-11	0.20	0.10	0.45	0.06
rs2008905	-0.36	6.81E-14	-	-	0.03	0.90
rs2271758	-0.22	1.34E-09	-0.41	9.13E-04	-	-
rs2787394	-0.28	8.64E-09	-	-	-0.69	3.71E-03
rs35337422	0.37	3.50E-08	-	-	0.12	0.71
rs4871827	-0.29	7.41E-09	-	-	-0.34	0.16
rs5442	-0.69	2.36E-13	-	-	-1.04	2.18E-02
rs62252355	-0.37	2.17E-16	-0.05	0.72	-	-
rs66511946	-0.36	2.15E-13	-	-	-0.70	1.07E-02
rs6989495	0.23	1.27E-09	-0.01	0.96	-	-
rs72739513	0.72	8.88E-09	-	-	0.26	0.71
rs7277632	0.34	1.20E-10	-	-	0.69	6.14E-03
rs73348111	1.12	7.15E-10	0.88	0.21	-	-
rs7503894	0.56	2.49E-29	-	-	0.38	0.14
rs79833181	0.86	1.55E-09	-0.06	0.92	-	-
rs9398171	0.50	7.51E-22	-	-	0.37	0.14
rs980772	-0.21	4.62E-08	-0.35	5.36E-03	-	-
