# Supplementary material for: Genetic variation affects morphological retinal phenotypes extracted from UK Biobank optical coherence tomography images
Source: PLoS Genet. 2021 May 12;17(5):e1009497. doi: 10.1371/journal.pgen.1009497 (PMC8143408; doi:10.1371/journal.pgen.1009497)
Supplement: S6 Table — LogMAR letters effect size refers to the difference in number of letters read on a standard LogMAR chart. A1 is the effect allele. (PDF) [file pgen.1009497.s006.pdf]

| SNP | A1 | A2 | Foveal thickness A-scan p-value | Foveal thickness B-scan p-value | LogMAR letters p-value | Foveal thickness A-scan effect size | Foveal thickness B-scan effect size | LogMAR letters effect size |
|---|---|---|---|---|---|---|---|---|
| rs7503894 | C | T | 1.26E-23 | 1.00E-25 | 3.54E-19 | -2.35 | -2.43 | -0.30 |
| rs1042602 | A | C | 1.23E-16 | 1.30E-16 | 3.61E-08 | 1.91 | 1.88 | 0.18 |
| rs1800407 | T | C | 3.87E-10 | 2.61E-10 | 2.16E-05 | 2.54 | 2.53 | 0.25 |
| rs17421627 | G | T | 1.36E-02 | 9.22E-03 | 0.06 | 1.05 | 1.09 | 0.07 |
| rs5442 | A | G | 0.07 | 4.35E-02 | 0.81 | 0.80 | 0.88 | 0.01 |
| rs9398171 | T | C | 0.06 | 0.05 | 0.96 | 0.47 | 0.47 | 0 |
| rs12998032 | C | T | 0.06 | 0.06 | 0.66 | 0.42 | 0.41 | 0.02 |
| rs79833181 | C | T | 0.14 | 0.07 | 0.70 | -1.32 | -1.57 | -0.02 |
| rs2008905 | T | C | 0.14 | 0.10 | 0.47 | -0.33 | -0.37 | 0.03 |
| rs13271359 | T | C | 0.14 | 0.11 | 0.23 | 0.38 | 0.40 | -0.05 |
| rs13215351 | T | A | 0.09 | 0.11 | 0.07 | -0.44 | -0.40 | -0.19 |
| rs12719025 | G | A | 0.16 | 0.14 | 0.64 | 0.32 | 0.33 | -0.02 |
| rs6989495 | T | G | 0.40 | 0.15 | 0.58 | -0.20 | -0.33 | 0.02 |
| rs146652416 | G | A | 0.33 | 0.21 | 0.73 | 0.68 | 0.87 | 0.04 |
| rs115520750 | T | G | 0.18 | 0.25 | 0.60 | 1.48 | 1.24 | -0.02 |
| rs1947075 | T | C | 0.40 | 0.27 | 0.75 | -0.20 | -0.25 | -0.04 |
| rs62252355 | C | T | 0.41 | 0.31 | 0.24 | -0.23 | -0.28 | -0.04 |
| rs17279437 | A | G | 0.31 | 0.31 | 0.80 | -0.36 | -0.36 | -0.01 |
| rs117304899 | G | C | 0.32 | 0.32 | 0.75 | -0.94 | -0.93 | 0.01 |
| rs7277632 | G | A | 0.16 | 0.33 | 0.98 | 0.35 | 0.24 | -0.02 |
| rs35337422 | C | A | 0.30 | 0.34 | 0.08 | 0.32 | 0.30 | -0.06 |
| rs181211282 | A | G | 0.42 | 0.38 | 0.07 | 0.58 | 0.63 | 0.08 |
| rs118031671 | G | T | 0.24 | 0.42 | 0.87 | 1.14 | 0.76 | 0 |
| rs72739513 | A | G | 0.64 | 0.44 | 0.58 | 0.28 | 0.45 | 0.08 |
| rs12574166 | T | C | 0.25 | 0.50 | 0.20 | -0.36 | -0.21 | 0.04 |
| rs73348111 | C | T | 0.44 | 0.50 | 0.34 | 0.86 | 0.74 | -0.13 |
| rs35001871 | C | G | 0.47 | 0.51 | 0.53 | 0.18 | 0.16 | -0.05 |
| rs980772 | T | G | 0.57 | 0.53 | 0.63 | -0.14 | -0.15 | -0.01 |
| rs376067714 | G | A | 0.71 | 0.53 | 0.05 | 0.12 | 0.21 | -0.12 |
| rs17095953 | A | G | 0.37 | 0.55 | 3.40E-02 | -0.24 | -0.16 | 0.08 |
| rs66511946 | G | A | 0.68 | 0.58 | 0.57 | -0.10 | -0.13 | -0.02 |
| rs2271758 | G | T | 0.58 | 0.58 | 0.50 | -0.12 | -0.12 | 0.09 |
| rs10140252 | T | G | 0.49 | 0.60 | 0.48 | 0.21 | 0.16 | 0.02 |
| rs4871827 | A | G | 0.67 | 0.64 | 0.67 | -0.10 | -0.11 | -0.03 |
| rs143330165 | T | C | 0.80 | 0.65 | 3.10E-02 | -0.29 | -0.51 | -0.37 |
| rs1470108 | A | C | 0.65 | 0.69 | 0.66 | -0.11 | -0.09 | -0.05 |
| rs2787394 | T | C | 0.77 | 0.70 | 0.09 | 0.06 | 0.08 | -0.25 |
| rs13010692 | C | T | 0.63 | 0.73 | 0.95 | -0.12 | -0.08 | 0 |
| rs10762201 | G | A | 0.59 | 0.75 | 0.14 | -0.14 | -0.08 | 0.09 |
| rs1254276 | T | C | 0.71 | 0.77 | 4.40E-02 | -0.08 | -0.06 | -0.07 |
| rs13083522 | G | A | 0.88 | 0.86 | 0.76 | -0.04 | -0.05 | -0.01 |
| rs11762530 | C | G | 0.97 | 0.89 | 0.40 | -0.01 | -0.03 | 0.04 |
| rs2004187 | C | A | 0.90 | 0.89 | 0.84 | -0.03 | 0.03 | 0 |
| rs149831820 | C | T | 0.95 | 0.93 | 0.17 | 0.03 | -0.04 | -0.06 |
| rs527871768 | A | G | 0.99 | 0.95 | 0.79 | 0.01 | -0.08 | 0.02 |
| rs117300236 | G | A | 0.97 | 0.96 | 1.10E-02 | 0.01 | 0.01 | -0.10 |
