# Supplementary material for: Genetic variation affects morphological retinal phenotypes extracted from UK Biobank optical coherence tomography images
Source: PLoS Genet. 2021 May 12;17(5):e1009497. doi: 10.1371/journal.pgen.1009497 (PMC8143408; doi:10.1371/journal.pgen.1009497)
Supplement: S7 Table — The 30 most significant associations (P<0.05) are listed. References [1] Pers, T. H. et al. Biological interpretation of genome-wide association studies using predicted gene functions. Nature Communications 6, 1–9 (2015). URL www.nature.com/naturecommunications. (PDF) [file pgen.1009497.s007.pdf]

Original gene set ID	Original gene set description	Nominal p-value	False discovery rate < 5%
MP:0003849	greasy coat	3.95E-06	No
REACTOME_SIGNALING_BY_INSULIN_RECEPTOR	REACTOME_SIGNALING_BY_INSULIN_RECEPTOR	2.94E-05	No
GO:0032862	activation of Rho GTPase activity	3.17E-05	No
REACTOME_IRS:MEDIATED_SIGNALLING	REACTOME_IRS:MEDIATED_SIGNALLING	4.66E-05	No
REACTOME_IRS:RELATED_EVENTS	REACTOME_IRS:RELATED_EVENTS	4.66E-05	No
GO:0032869	cellular response to insulin stimulus	7.87E-05	No
GO:0032856	activation of Ras GTPase activity	9.05E-05	No
ENSG00000167283	ATP5L subnetwork	1.02E-04	No
ENSG00000147082	CCNB3 subnetwork	1.23E-04	No
GO:0008286	insulin receptor signaling pathway	1.29E-04	No
ENSG00000080845	DLGAP4 subnetwork	1.93E-04	No
ENSG00000141252	VPS53 subnetwork	3.24E-04	No
ENSG00000197442	MAP3K5 subnetwork	3.59E-04	No
GO:0071482	cellular response to light stimulus	3.60E-04	No
GO:0034394	protein localization at cell surface	3.90E-04	No
KEGG_ENDOMETRIAL_CANCER	KEGG_ENDOMETRIAL_CANCER	4.90E-04	No
REACTOME_INSULIN_RECEPTOR_SIGNALLING_CASCADE	REACTOME_INSULIN_RECEPTOR_SIGNALLING_CASCADE	5.44E-04	No
ENSG00000173744	AGFG1 subnetwork	6.74E-04	No
GO:0032868	response to insulin stimulus	6.92E-04	No
GO:0032386	regulation of intracellular transport	8.10E-04	No
GO:0055015	ventricular cardiac muscle cell development	8.57E-04	No
GO:0071375	cellular response to peptide hormone stimulus	1.25E-03	No
MP:0001304	cataracts	1.31E-03	No
GO:0009925	basal plasma membrane	1.34E-03	No
GO:0004712	protein serine/threonine/tyrosine kinase activity	1.36E-03	No
GO:0071900	regulation of protein serine/threonine kinase activity	1.36E-03	No
REACTOME_PI3K_CASCADE	REACTOME_PI3K_CASCADE	1.46E-03	No
MP:0001382	abnormal nursing	1.48E-03	No
MP:0000167	decreased chondrocyte cell number	1.51E-03	No
ENSG00000163558	PRKCI subnetwork	1.59E-03	No
