# Supplementary material for: Genetic variation affects morphological retinal phenotypes extracted from UK Biobank optical coherence tomography images
Source: PLoS Genet. 2021 May 12;17(5):e1009497. doi: 10.1371/journal.pgen.1009497 (PMC8143408; doi:10.1371/journal.pgen.1009497)
Supplement: S8 Table — All nominally significant associations (P<0.05) are listed. References [1] Pers, T. H. et al. Biological interpretation of genome-wide association studies using predicted gene functions. Nature Communications 6, 1–9 (2015). URL www.nature.com/naturecommunications. (PDF) [file pgen.1009497.s008.pdf]

MeSH term	MeSH first level term	MeSH second level term	Nominal p-value	False discovery rate < 5%
A09.371	Eye	Sense Organs	3.86E-03	No
A03.556.875.875	Stomach	Digestive System	5.57E-03	No
A03.556.875	Upper Gastrointestinal Tract	Digestive System	6.03E-03	No
A03.556.500.760.464	Parotid Gland	Digestive System	0.01	No
A03.556.500.760	Salivary Glands	Digestive System	0.01	No
A07.541.510	Heart Valves	Cardiovascular System	0.02	No
A07.541.510.110	Aortic Valve	Cardiovascular System	0.02	No
A03.556.875.500	Esophagus	Digestive System	0.02	No
A05.360.319.887	Vulva	Urogenital System	0.03	No
A14.549	Mouth	Stomatognathic System	0.03	No
A10.615.550.599	Mouth Mucosa	Tissues	0.03	No
A03.556	Gastrointestinal Tract	Digestive System	0.05	No
