# Supplementary material for: Genetic variation affects morphological retinal phenotypes extracted from UK Biobank optical coherence tomography images
Source: PLoS Genet. 2021 May 12;17(5):e1009497. doi: 10.1371/journal.pgen.1009497 (PMC8143408; doi:10.1371/journal.pgen.1009497)
Supplement: S9 Table — Values reported for two meta-analysis methods, MR Egger and Inverse Variance Weighted. POAG summary statistics were taken from the POAG International Glaucoma Genetics Consortium (IGGC) meta-analysis [1]. Summary statistics for genetic association studies of IOP, were taken from [2]. References [1] Gharahkhani, P. et al. Genome-wide meta-analysis identifies 127 open-angle glaucoma loci with consistent effect across ancestries. Nature Communications 12 (2021). URL https://pubmed.ncbi.nlm.nih.gov/33627673/. [2] Khawaja, A. P. et al. Genome-wide analyses identify 68 new loci associated with intraocular pressure and improve risk prediction for primary open-angle glaucoma. Nature Genetics 50, 778–782 (2018). (PDF) [file pgen.1009497.s009.pdf]

Exposure	Outcome	MR Egger			Inverse Variance Weighted		
		Effect size	P-value	SE	Effect size	P-value	SE
IOP	POAG	0.66	1.68E-14	0.07	0.55	0.03	2.80E-85
GCIPL	POAG	-4.39E-03	0.92	0.05	0.01	0.50	0.02
GCIPL	IOP	4.70E-03	0.94	0.07	-7.93E-03	0.73	0.02
RNFL	POAG	-0.01	0.91	0.09	-0.04	0.18	0.03
RNFL	IOP	-0.08	0.26	0.07	-7.26E-03	0.77	0.02
GCIPL and RNFL meta	POAG	0.02	0.66	0.05	-0.01	0.58	0.02
GCIPL and RNFL meta	IOP	-0.03	0.56	0.04	0.01	0.76	0.02
POAG	GCIPL	-0.53	0.02	0.22	-0.16	0.06	0.09
POAG	RNFL	-0.41	0.03	0.18	-0.12	0.09	0.07
POAG	GCIPL and RNFL meta	-0.72	3.62E-03	0.24	-0.22	0.02	0.10
IOP	GCIPL	-0.08	0.65	0.18	-0.06	0.40	0.07
IOP	RNFL	-0.12	0.38	0.13	0.05	0.30	0.05
IOP	GCIPL and RNFL meta	-0.25	0.19	0.19	0.01	0.91	0.75
