# Supplementary figures and images for: Genetic variation affects morphological retinal phenotypes extracted from UK Biobank optical coherence tomography images
Source: PLoS Genet. 2021 May 12;17(5):e1009497. doi: 10.1371/journal.pgen.1009497 (PMC8143408; doi:10.1371/journal.pgen.1009497)

A

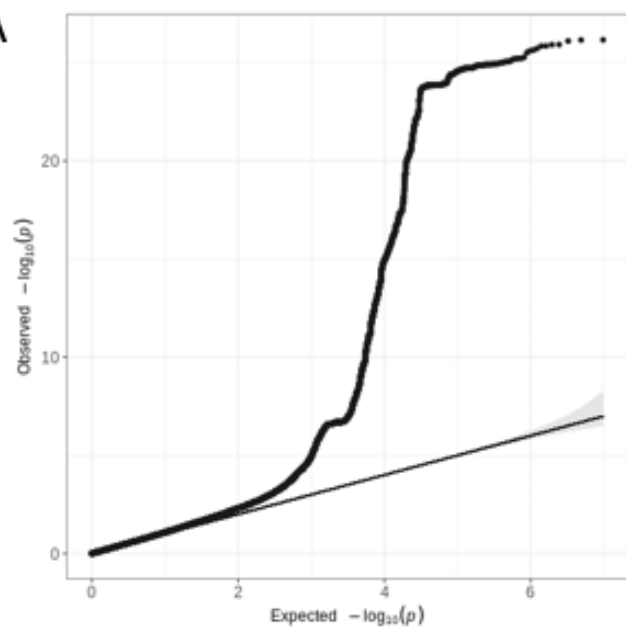

B

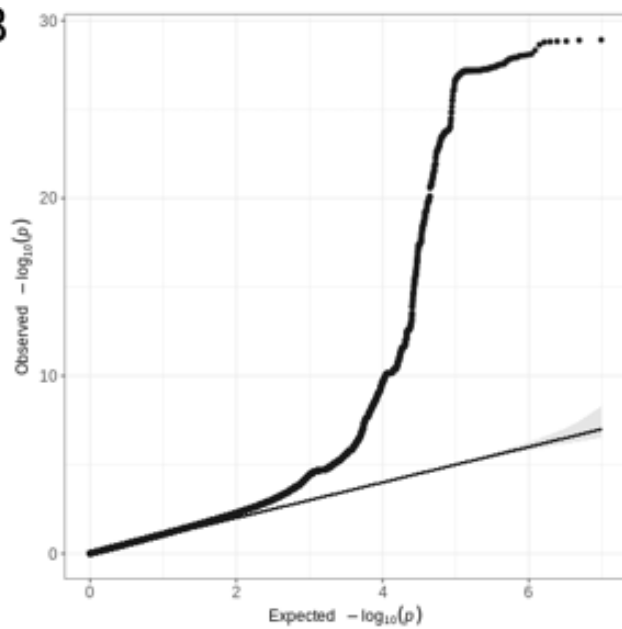

Supplement: S1 Fig — (A) The quantile-quantile plot (qq-plot) for the GWAS of RNFL thickness prior to meta-analysis (Lambda GC = 1.11, Intercept = 1.01, Ratio = 0.05). (B) The qq-plot for the GWAS of GCIPL thickness prior to meta-analysis (Lambda GC = 1.12, Intercept = 1.01, Ratio = 0.05). (PDF) [file pgen.1009497.s010.pdf]

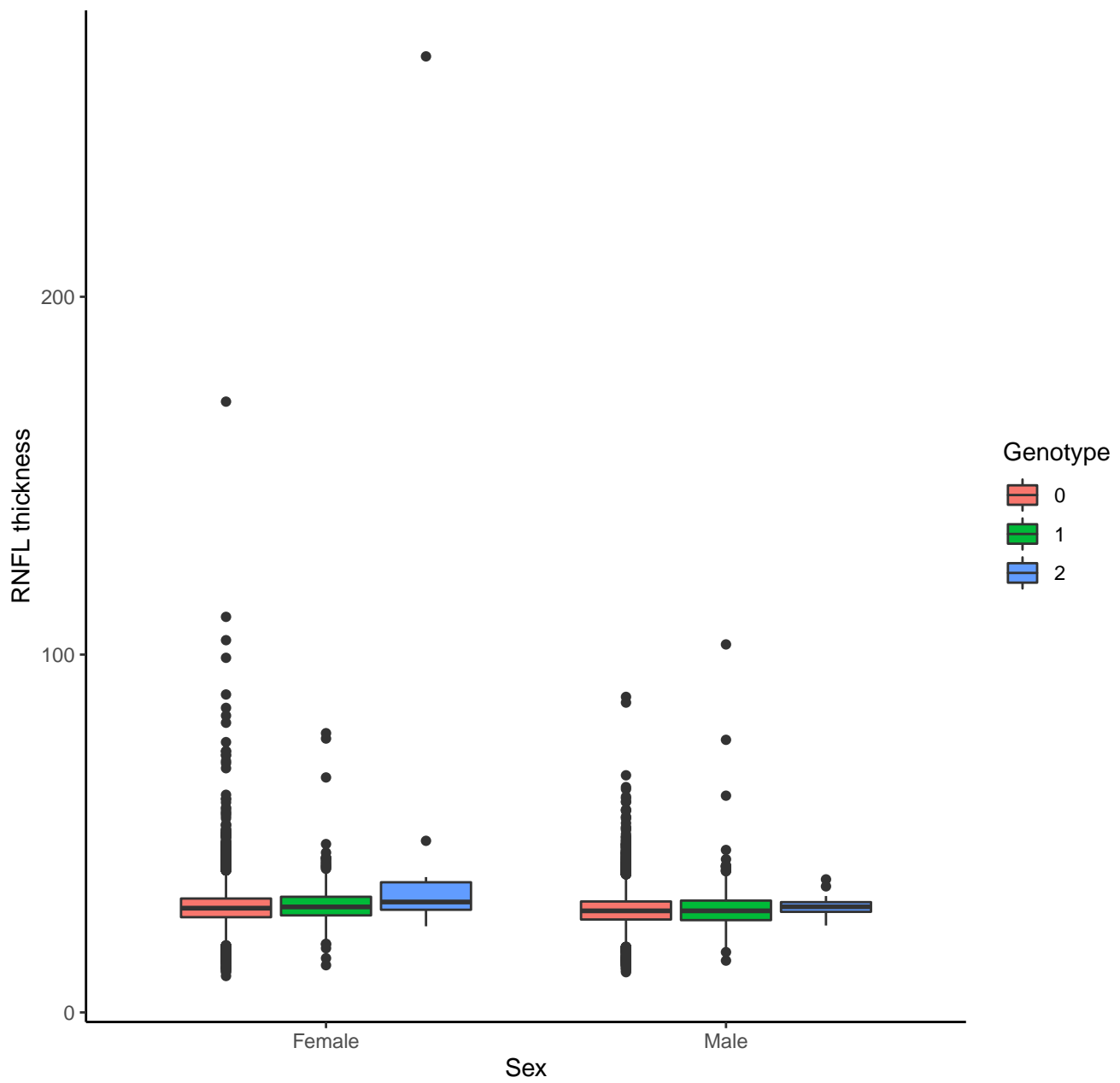

Supplement: S2 Fig — A boxplot of the mean thickness of the RNFL across the Macula 6 gird and across the left and right eyes stratified by genetically determined sex and genotype at rs146652416 (FOXG1). Genotype is encoded as homozygous reference (0), heterozygous (1) and homozygous alternative (2). A linear model of RNFL thickness including each discovered SNP and an interaction term between the SNP and genetically determined sex had a statistically significant sex-specific effect for rs146652416 (P = 8.08 × 10^-4^). (PDF) [file pgen.1009497.s011.pdf]

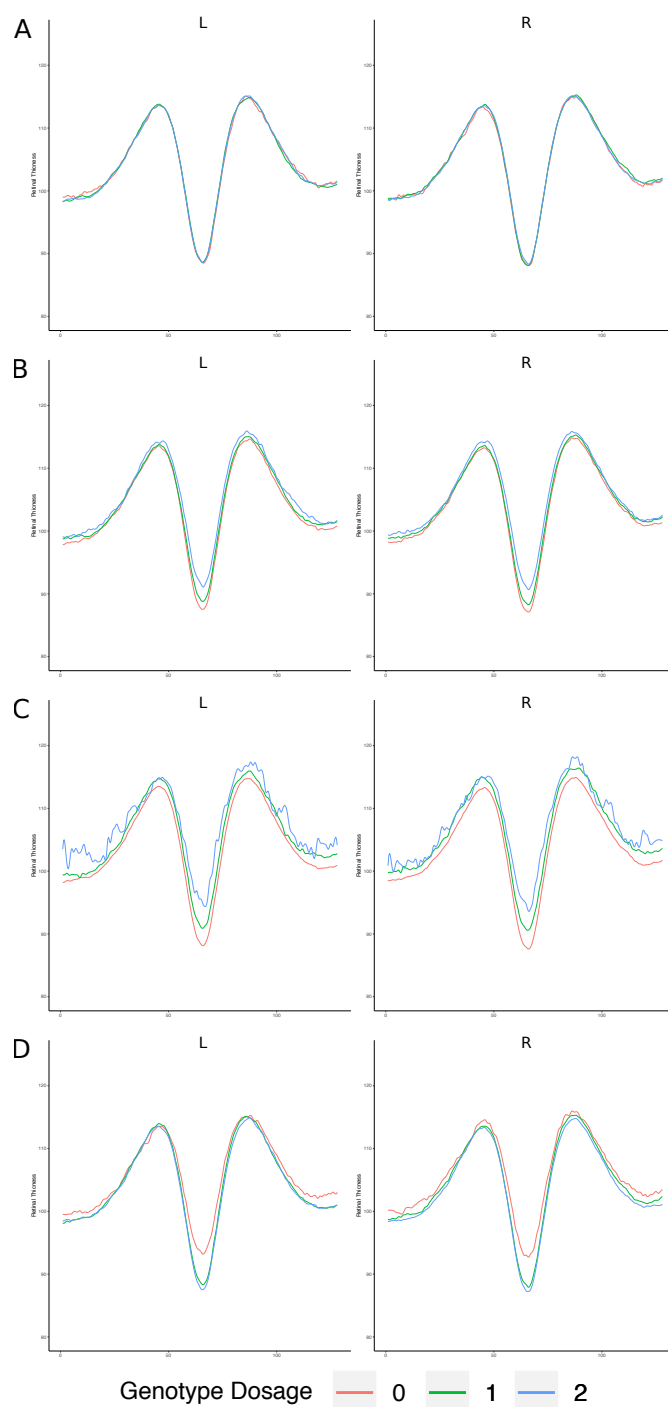

Supplement: S3 Fig — Mean cross-sectional models of overall retinal thickness across three groups defined by their allele state, homozygous reference, heterozygous or homozygous alternative at: (A) IGFBP3 (rs11762530), the variant with the lowest P-value aside from TSPAN10, as a control (B) TYR (rs1042602) (C) OCA2 (rs1800407) (D) TSPAN10 (rs7503894). The y axis is representative of total retinal thickness. (PDF) [file pgen.1009497.s012.pdf]

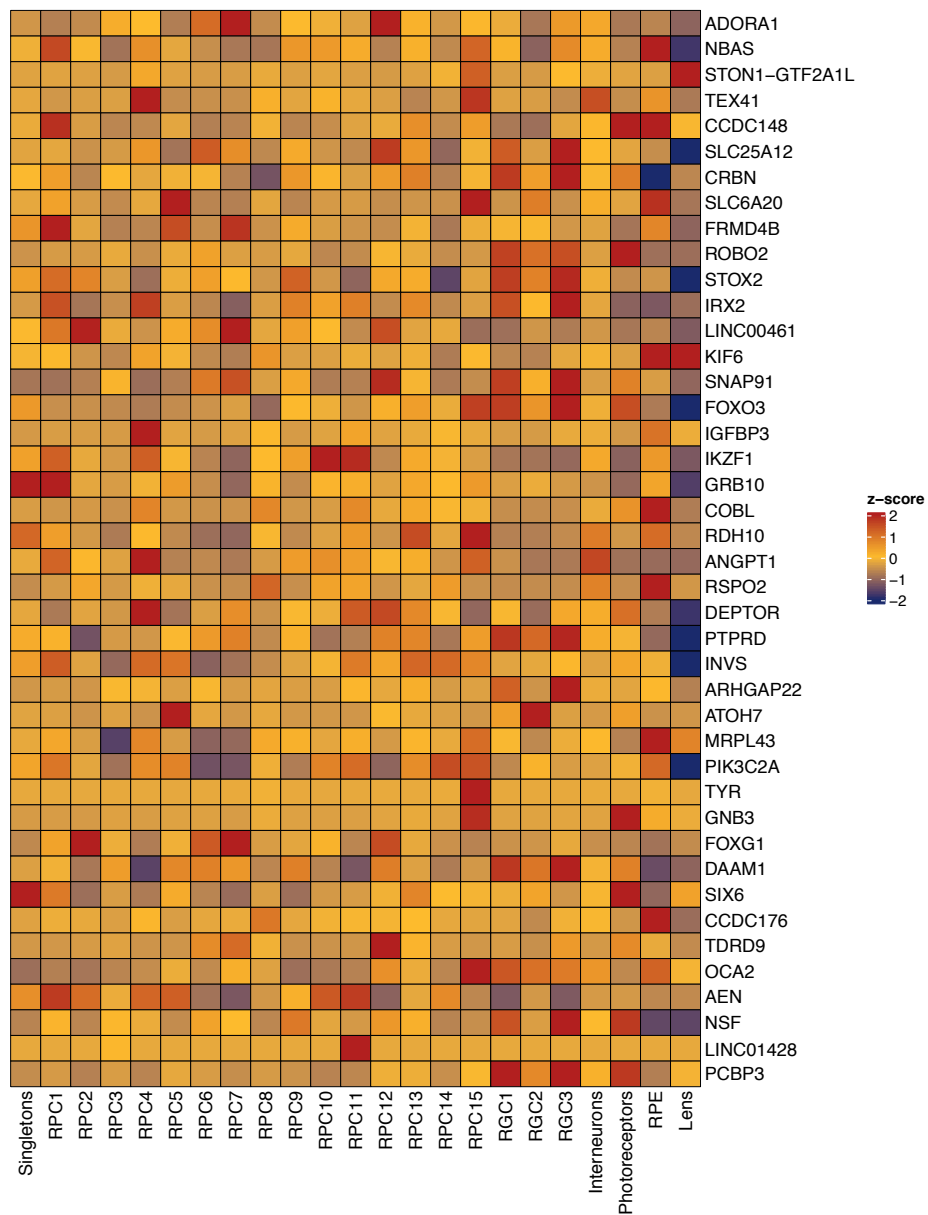

Supplement: S4 Fig — A heatmap of the mean expression of the genes labelled by the loci associated with inner retinal thickness in the different retinal cell subpopulations: singletons, retinal progenitor cells (RPC), retinal ganglion cells (RGC), interneurons, photoreceptors, retinal pigment epithelium (RPE) and lens. Expression values have been scaled and converted to z-scores. (PDF) [file pgen.1009497.s013.pdf]

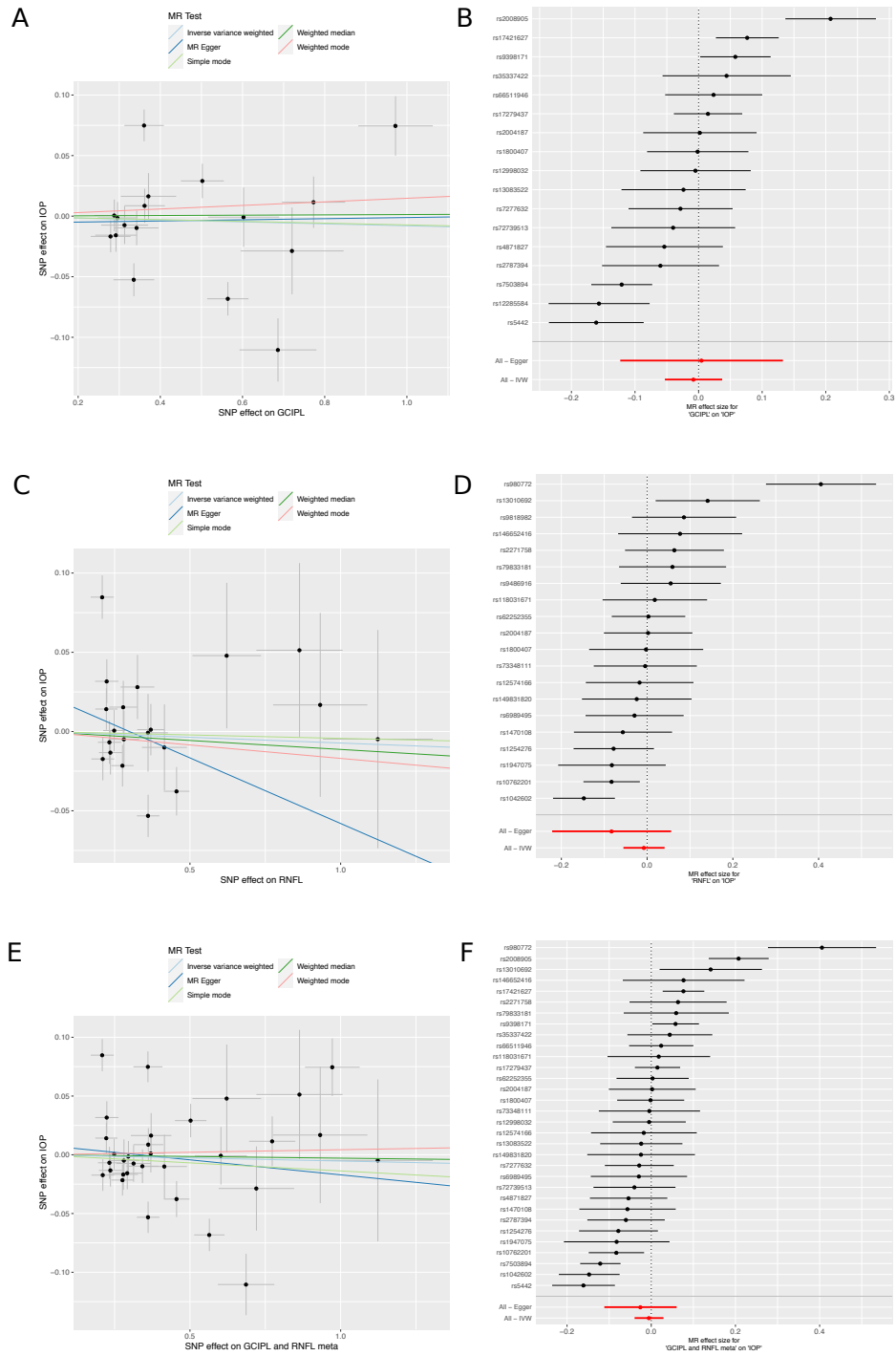

Supplement: S5 Fig — (A) Scatter plot of the relationship between the effect size of SNPs found significantly associated with GCIPL thickness, and the effect size of those SNPs on IOP. (B) Forest plot showing the effect size and direction of SNPs significantly associated with GCIPL thickness on IOP. (C) Scatter plot of the relationship between the effect size of SNPs found significantly associated with RNFL thickness, and the effect of those SNPs on IOP. (D) Forest plot showing the effect size and direction of SNPs significantly associated with RNFL thickness on IOP. (E) Scatter plot of the relationship between the effect size of SNPs found significantly associated in the meta-analysed GCIPL and RNFL thicknesses, and the effect of those SNPs on IOP. (F) Forest plot showing the effect size and direction of SNPs significantly associated in the meta-analysed GCIPL and RNFL thickness on IOP. Summary statistics for genetic association studies of IOP, were taken from [1]. References [1] Khawaja, A. P. et al. Genome-wide analyses identify 68 new loci associated with intraocular pressure and improve risk prediction for primary open-angle glaucoma. Nature Genetics 50, 778–782 (2018). (PDF) [file pgen.1009497.s014.pdf]

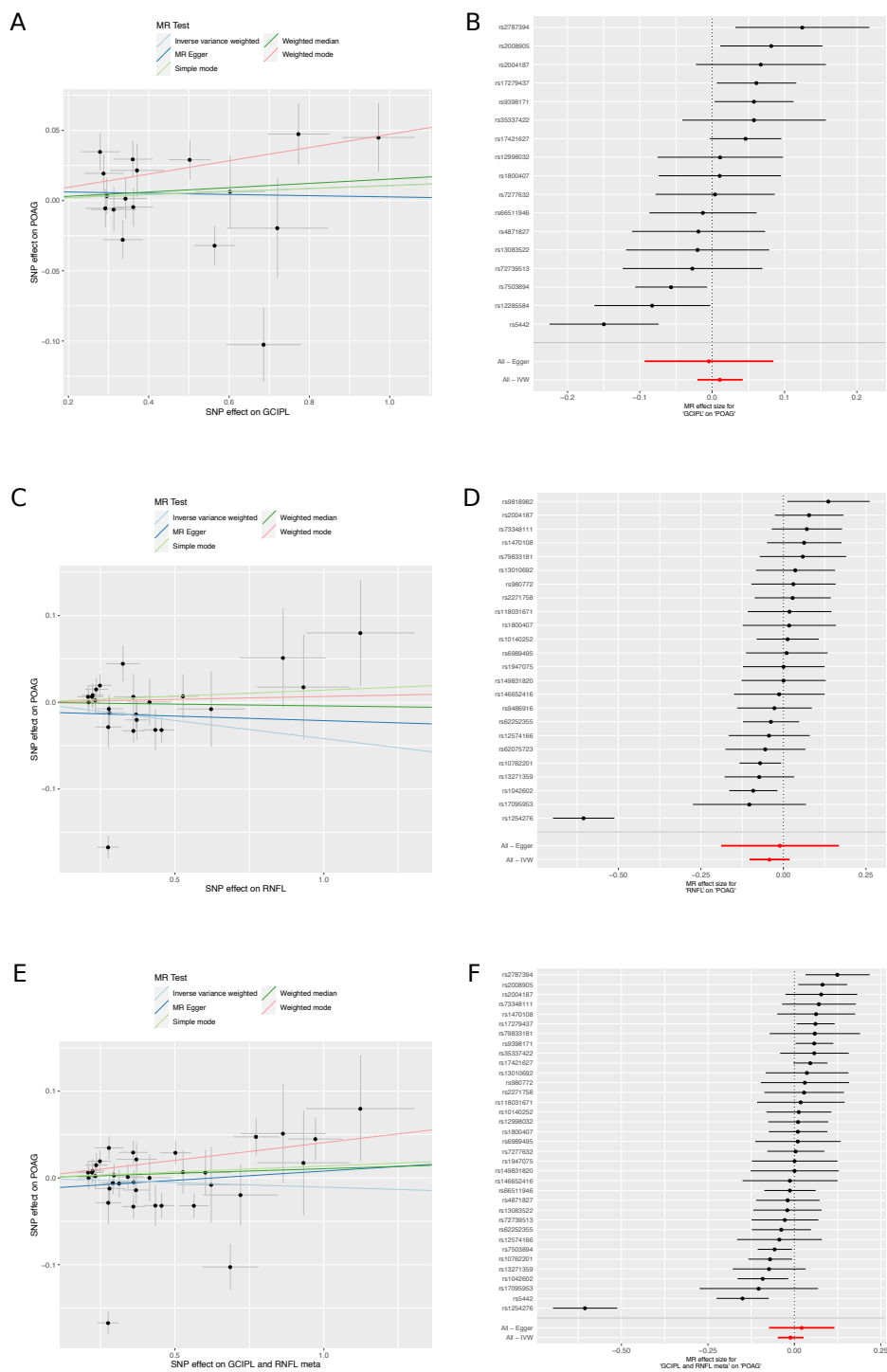

Supplement: S6 Fig — (A) Scatter plot of the relationship between the effect size of SNPs found significantly associated with GCIPL thickness, and the effect size of those SNPs on POAG. (B) Forest plot showing the effect size and direction of SNPs significantly associated with GCIPL thickness on POAG. (C) Scatter plot of the relationship between the effect size of SNPs found significantly associated with RNFL thickness, and the effect of those SNPs on POAG. (D) Forest plot showing the effect size and direction of SNPs significantly associated with RNFL thickness on POAG. (E) Scatter plot of the relationship between the effect size of SNPs found significantly associated in the meta-analysed GCIPL and RNFL thicknesses, and the effect of those SNPs on POAG. (F) Forest plot showing the effect size and direction of SNPs significantly associated in the meta-analysed GCIPL and RNFL thickness on POAG. POAG summary statistics were taken from the POAG International Glaucoma Genetics Consortium (IGGC) meta-analysis [1]. Refrences [1] Gharahkhani, P. et al. Genome-wide meta-analysis identifies 127 open-angle glaucoma loci with consistent effect across ancestries. Nature Communications 12 (2021). URL https://pubmed.ncbi.nlm.nih.gov/33627673/. (PDF) [file pgen.1009497.s015.pdf]

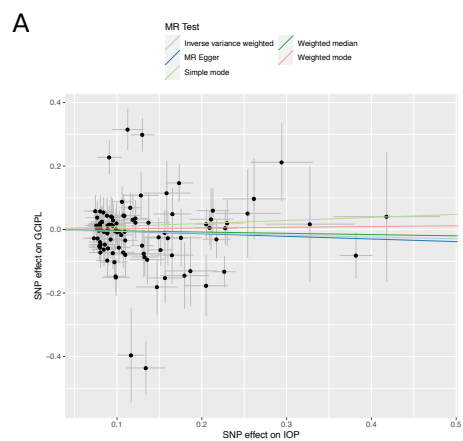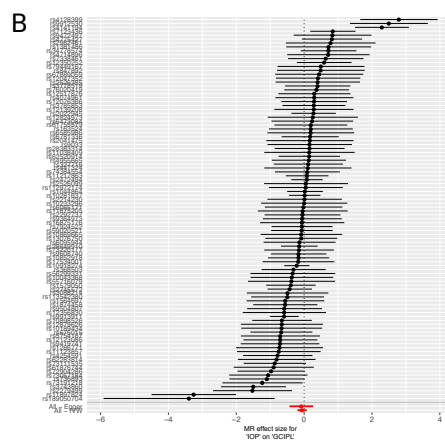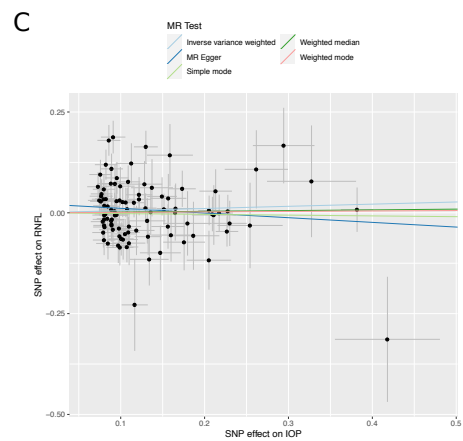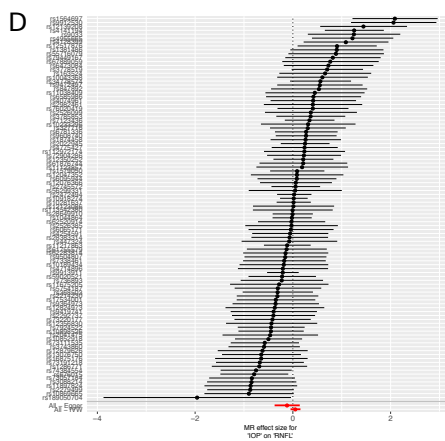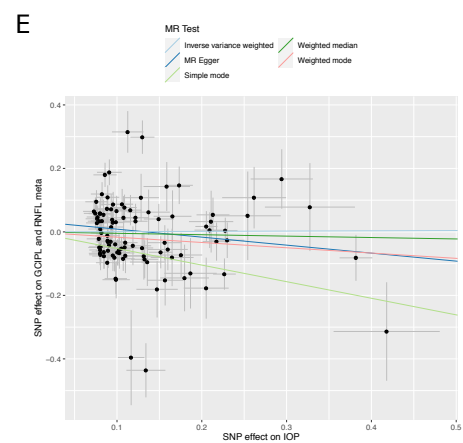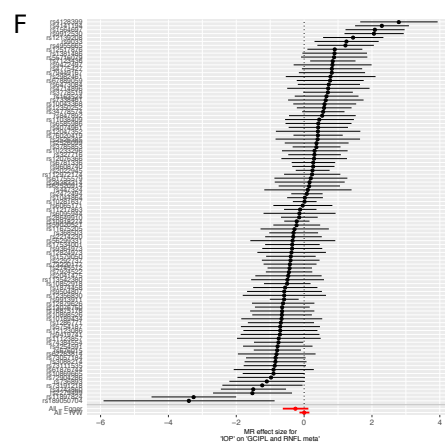

Supplement: S7 Fig — (A) Scatter plot of the relationship between the effect size of SNPs found significantly associated with IOP, and the effect size of those SNPs on GCIPL thickness. (B) Forest plot showing the effect size and direction of SNPs significantly associated with IOP on GCIPL thickness. (C) Scatter plot of the relationship between the effect size of SNPs found significantly associated with IOP, and the effect of those SNPs on RNFL thickness. (D) Forest plot showing the effect size and direction of SNPs significantly associated with IOP on RNFL thickness. (E) Scatter plot of the relationship between the effect size of SNPs found significantly associated with IOP and the effect size of those SNPs in the meta-analysed GCIPL and RNFL thickness. (F) Forest plot showing the effect size and direction of SNPs significantly associated with IOP on the meta-analysed GCIPL and RNFL thickness. Summary statistics for genetic association studies of IOP, were taken from [1]. References [1] Khawaja, A. P. et al. Genome-wide analyses identify 68 new loci associated with intraocular pressure and improve risk prediction for primary open-angle glaucoma. Nature Genetics 50, 778–782 (2018). (PDF) [file pgen.1009497.s016.pdf]

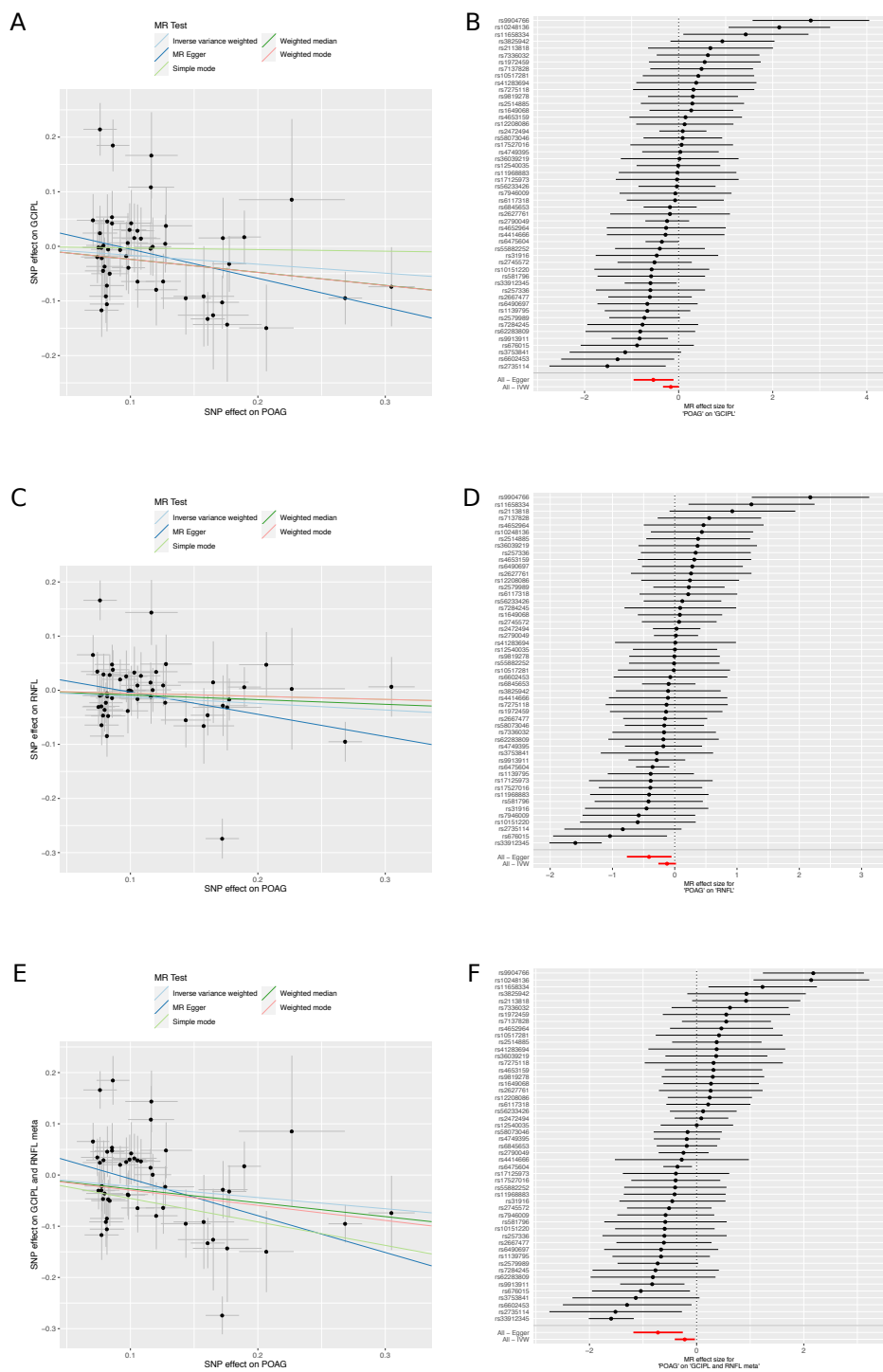

Supplement: S8 Fig — (A) Scatter plot of the relationship between the effect size of SNPs found significantly associated with POAG, and the effect size of those SNPs on GCIPL thickness. (B) Forest plot showing the effect size and direction of SNPs significantly associated with POAG on GCIPL thickness. (C) Scatter plot of the relationship between the effect size of SNPs found significantly associated with POAG, and the effect of those SNPs on RNFL thickness. (D) Forest plot showing the effect size and direction of SNPs significantly associated with POAG on RNFL thickness. (E) Scatter plot of the relationship between the effect size of SNPs found significantly associated with POAG and the effect size of those SNPs in the meta-analysed GCIPL and RNFL thickness. (F) Forest plot showing the effect size and direction of SNPs significantly associated with POAG on the meta-analysed GCIPL and RNFL thickness. POAG summary statistics were taken from the POAG International Glaucoma Genetics Consortium (IGGC) meta-analysis [1]. Refrences [1] Gharahkhani, P. et al. Genome-wide meta-analysis identifies 127 open-angle glaucoma loci with consistent effect across ancestries. Nature Communications 12 (2021). URL https://pubmed.ncbi.nlm.nih.gov/33627673/. (PDF) [file pgen.1009497.s017.pdf]

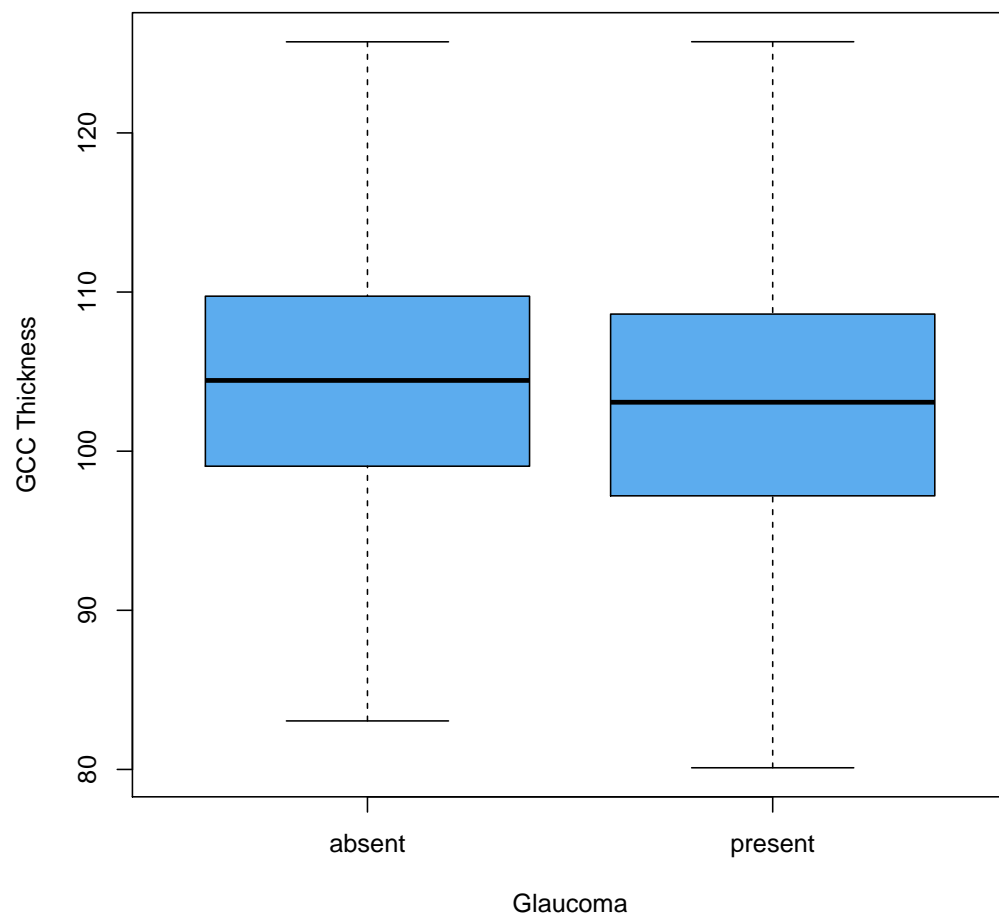

Supplement: S9 Fig — A boxplot showing the thickness of the ganglion cell complex in the dataset comparing those who have glaucoma (n = 2,751), to those that do not (n = 28,314). An accompanying Wilcoxon rank sum test was performed that showed a statistically significant difference between the two populations (P = 2.2 × 10^-16^). (PDF) [file pgen.1009497.s018.pdf]
